# Improvement of the phytoremediation efficiency of *Neyraudia reynaudiana* for lead-zinc mine-contaminated soil under the interactive effect of earthworms and EDTA

**DOI:** 10.1038/s41598-018-24715-2

**Published:** 2018-04-23

**Authors:** Ying Li, Jiewen Luo, Jiaoda Yu, Lidan Xia, Chuifan Zhou, Liping Cai, Xiangqing Ma

**Affiliations:** 1 College of Forestry Fujian Agriculture and Forestry University, Fuzhou, China; 2 Co-Innovation Center For Soil and Water Conservation in Red Soil Region of the Cross-Straits, Fuzhou, China; 3 Red Soil Hilly Ecosystem Positioning Observation Station in Changting of Fujian, Fuzhou, China

## Abstract

Slow plant growth, low biomass, and low bioavailability of heavy metals in soil are important factors that limit remediation efficiencies. This study adopted a pot cultivation method to evaluate the phytoremediation efficiency of *Neyraudia reynaudiana*, planted in contaminated soil from a lead-zinc mining area. The soil was inoculated with earthworms (*Eisenia fetida*), and mixed with the chelating agent ethylenediaminetetraacetic acid (EDTA) one month after planting. The addition of earthworms significantly increased the aboveground biomass of *N. reynaudiana* and activated heavy metals in the soil, thus facilitating heavy metal uptake by *N. reynaudiana*. The addition of EDTA significantly increased the incorporation and transport of heavy metals, reduced the uptake of heavy metals by the plant cell wall, and increased the proportions of cellular soluble constituents. Especially with regard to lead, inoculation with earthworms and EDTA application significantly promoted the accumulation efficiency of *N. reynaudiana*, increasing it 7.1-16.9-fold compared to the control treatment without earthworms and EDTA, and 1.5-2.3-fold compared to a treatment that only used EDTA.

## Introduction

*Neyraudia reynaudiana* is a pioneer plant that is widely distributed in abandoned lead-zinc (Pb-Zn) mining areas that are subjected to high heavy metal contents, drought conditions, and nutrient depletion in Fujian, China. *Neyraudia reynaudiana* is highly tolerant to the conditions of lead-zinc mining areas and readily accumulates Pb from the soil^1^. Based on field investigations, the achieved Pb content in the aerial part of *N. reynaudiana* is as high as 345–773 mg/kg. Although this species is not a hyperaccumulator for Pb, *N. reynaudiana* has a larger biomass (plant height about 300 cm, diameter of roughly 1 cm, and well-developed roots as long as 300 cm) compared to short and slow-growing hyperaccumulators (Fig. S1); *N. reynaudiana* also grows rapidly in resource-poor and harsh environments. These features make it a suitable remediation plant for Pb contaminated soil in the Pb-Zn mining area of Fujian as well as an adequate greening plant that is used for soil reinforcement and slope protection along heavy metal contaminated roads^1^.

The successful application of phytoremediation techniques is limited by three factors: slow growth and low biomass of accumulator plants and low bioavailability of heavy metals in soil^2–4^. Some earthworm species, such as *Eisenia foetida*, *Lumbricus terrestris*, *Lumbricus rubellus*, or *Aporrectodea caliginosa*, can survive in soils that are polluted with heavy metals and even accumulate heavy metals such as Pb, Cd, Zn, and Cu^5,6^. Moreover, previous studies indicated that soil inoculation with earthworms can significantly improve soil structure, aeration, permeability, and available nutrients^5–7^. In addition, earthworms are also “mixers” and “transmitters” of soil organic matter and microorganisms, which either directly or indirectly increases plant growth rates^7^. In this context, the introduction of earthworms into heavy metal contaminated soils has been suggested to complement phytoremediation programs. According to a previous study, *Eisenia foetida* can promote the activation of heavy metals in sludge and consequently improve the fertility of sludge^8^. In addition, after inoculating As-contaminated agricultural soils with *Eisenia foetida*, earthworm activities significantly increased aboveground and belowground *Zea mays* biomass, irrespective of the As concentration^9,10^. To improve the bioavailability of heavy metals in contaminated soil, addition of chelating agents (e.g., low-molecular organic acids, ethylenediaminetetraacetic acid (EDTA), ethylenediamine-N, N’-disuccinic acid (EDDS), humic substances, and nitrilotriacetic acid) is a common method in phytoremediation and has been suggested to mobilize heavy metals in the soil, improve their bioavailability, and promote plant uptake^11–14^. EDTA in particular can chelate with metallic ions to form the most stable chelates, thus resulting in the desorption of heavy metals, the prevention precipitation, and the increase of plant uptake of heavy metals^10–17^. Based on previous studies, EDTA has been shown to have a high binding constant for metals (Cu, 20.49; Ni, 20.11; Pb, 19.71; Co, 18.1; Cd, 18.10; Zn, 18.00), allowing the dissolution of heavy metals^13,14^. EDTA addition may increase metal solubilization by up to 600-fold, depending on the type of metal and the soil conditions^11^. Luo *et al*.^12^ reported that the addition of 5 mmol kg^−1^ of EDTA was most effective for the accumulation of Pb by *Chrysanthemum*, achieving levels 27.1 times higher than the control treatment. Grcman *et al*.^15^ reported that the addition of 10 mmol kg^−1^ of EDTA to soil increased the concentrations of Pb, Zn, and Cd in *Brassica rapa* roots by 41, 71, and 69%, respectively, compared to the control group. However, the accumulation of Pb, Zn, and Cd in the *B. rapa* shoots accounted for 37.9, 10.4, and 56.3% of the total amounts in the soil, respectively. These results indicate that EDTA can accelerate soil remediation by plants.

Consequently, we tested the following hypotheses: i) earthworms can increase *N. reynaudiana* biomass in contaminated soil; ii) subsequent addition of EDTA can further increase the heavy metal accumulation by *N. reynaudiana*. The objectives of our study were therefore: i) to evaluate the change of heavy metal forms that are present in soil, ii) to compare the difference in *N. reynaudiana* biomass between contaminated and uncontaminated soils, and iii) to assess the accumulating effect for heavy metals in *N. reynaudiana* for sole and combined application of earthworms and EDTA.

## Results and Analysis

### Alteration of *N. reynaudiana* biomass under the interactive effects of earthworms and EDTA

**Figure 1:**
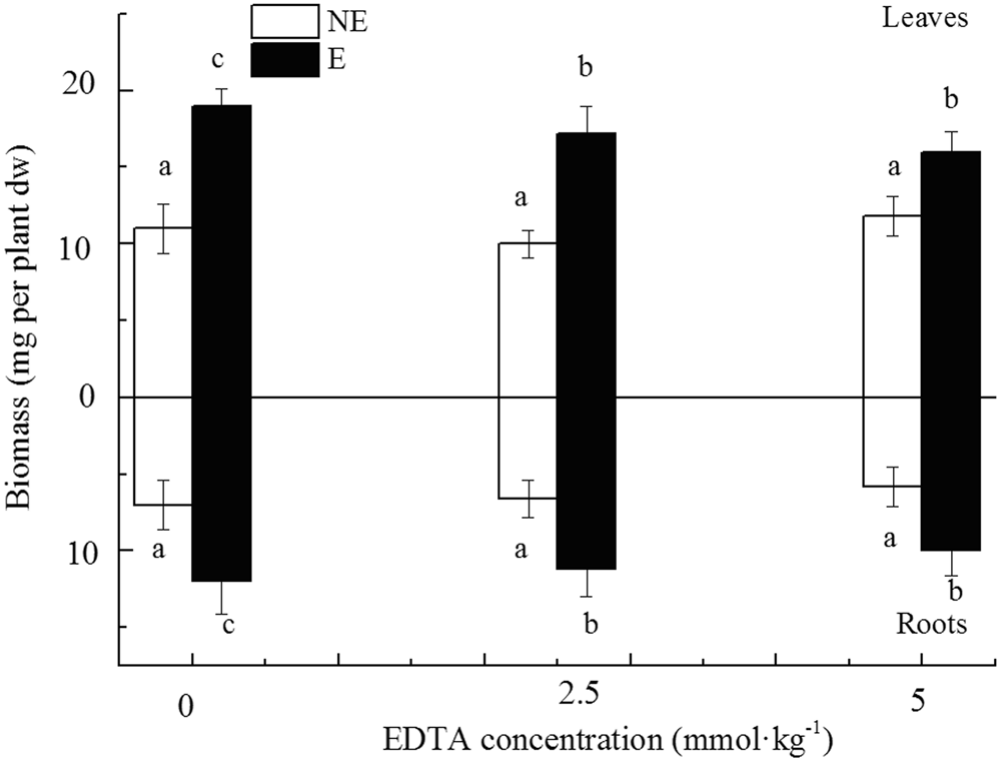
Changes of *N. reynaudiana* biomass under different concentrations of EDTA with (E) or without earthworms (NE).Values followed by different letters are significantly different at *P* < 0.05, as determined via SNK multiple range tests.

Most of the earthworms (83%) survived treatment without EDTA (see Table S1). However, EDTA addition significantly increased earthworm mortality and heavy metal absorption (Table S1, Fig. S3). The addition of earthworms significantly increased both above- and belowground biomass of *N. reynaudiana*. Compared to treatments without earthworms, above- and belowground biomass of *N. reynaudiana* increased 1.5-1.7-fold, while EDTA addition inhibited *N. reynaudiana* growth (Fig. 1).

### Heavy metal accumulation by *N. reynaudiana* under the interactive effects of earthworms and EDTA

**Figure 2:**
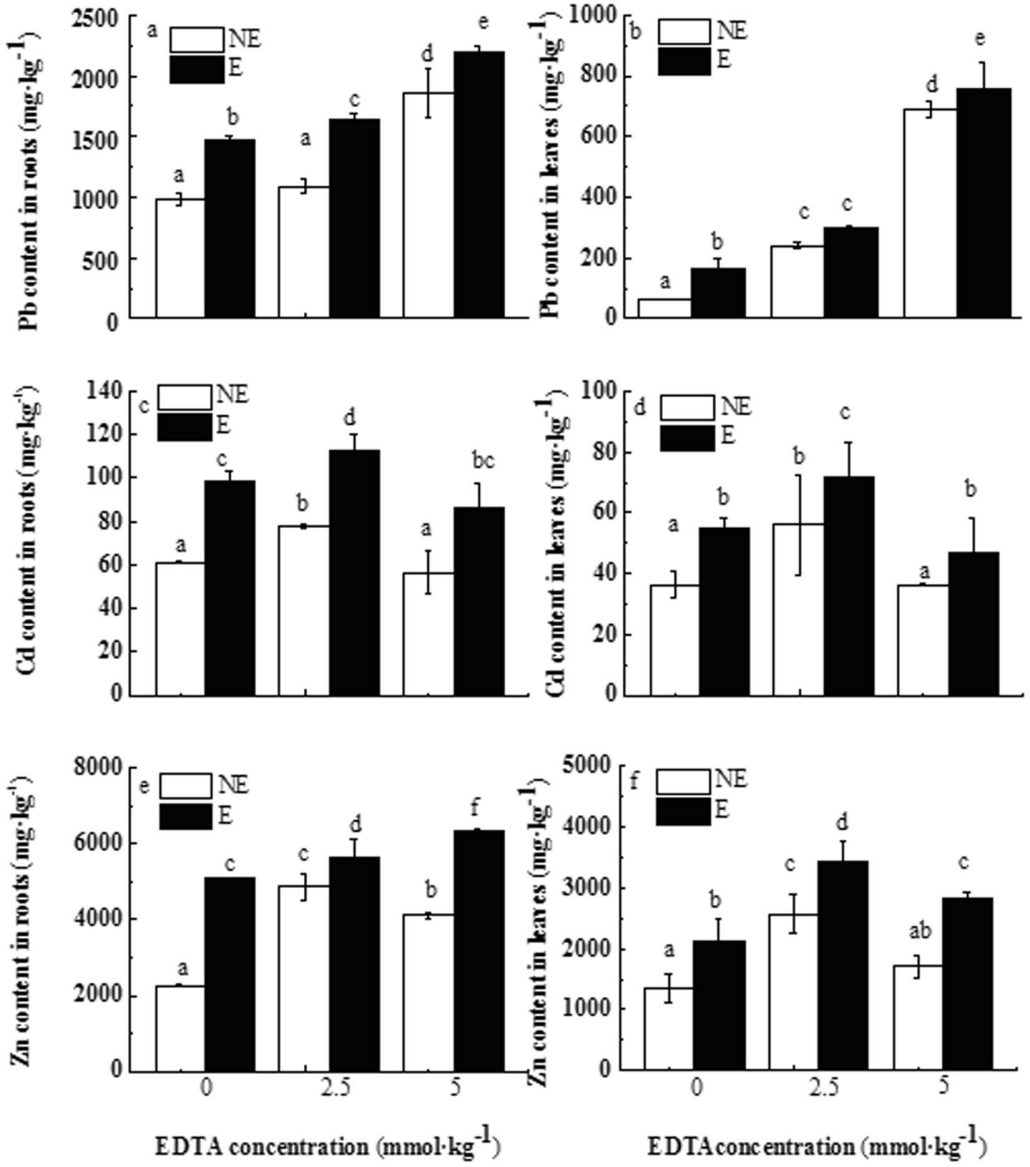
Changes of heavy metal accumulation in *N. reynaudiana* under different concentrations of EDTA with (E) or without earthworms (NE) (mg kg^−1^). Notes: different upper- and lowercase letters represent a significant difference between E and NE treatments under different concentrations (*P* < 0.05), as determined via SNK multiple range tests. The asterisk denotes a significance differences between E and NE treatments under the same EDTA concentrations (*P < 0.1; **P < 0.05; and ***P < 0.01), as determined via paired samples t-test.

Concentrations of Pb, Cd, and Zn in *N. reynaudiana* roots and leaves, as well as the total accumulation for single plants in treatments with earthworms, were both higher than those in treatments without earthworms. Without EDTA application, the Pb accumulations of *N. reynaudiana* leaves and roots was 2.6 times and 1.5 times higher than that of the control group (the accumulations of single plant’s roots and leaves were 4.5 times and 2.6 times higher than that in the control group without earthworms), respectively (Fig. 2, Fig. S2). This indicates that earthworm inoculation can noticeably increase the uptake and transport of Pb from the soil environment to the plant biomass by *N. reynaudiana*.

With increasing EDTA concentrations (with or without earthworms), the Pb content in *N. reynaudiana* roots and leaves, total Pb accumulation, as well as BCF, TF, and EE levels all increased (Table S2). In response to the treatment with 5 mmol·L^−1^ EDTA, the Pb content of *N. reynaudiana* roots and leaves was highest. In treatments without earthworms, the maximum Pb contents in *N. reynaudiana* leaves and roots were 688.12 and 1,852.49 mg·kg^−1^, respectively (total accumulations of single plants was 8.1 mg and 10.7 mg, respectively). These values were 10.69 and 1.88 times higher than in the control treatment without EDTA and earthworms (total accumulations of single plants were 11.4 and 1.5 times higher than those of the control group). In treatments with earthworms, the maximum Pb contents in *N. reynaudiana* leaves and roots were 757.70 and 2,193.93 mg·kg^−1^, respectively (total accumulations of single plants were 12.12 mg and 21.93 mg, respectively), which was 11.8 and 2.2 times higher than the values of the control treatment without EDTA and earthworms (total accumulations of single plants were 1.7 and 3.2 times higher than that of the control group without EDTA and earthworms).

The Cd and Zn uptake by *N. reynaudiana* showed patterns that differed from those observed for Pb uptake. With increasing EDTA concentrations, the Cd and Zn uptake of *N. reynaudiana* first increased and then decreased; under earthworm inoculation and for an EDTA concentration of 2.5 mmol·L^−1^, Cd and Zn uptake reached maximum values, accounting for 71.8 mg·kg^−1^ for leaves and 112.2 mg·kg^−1^ for and roots (the total accumulations of single plants were 1.26 and 1.24 mg, respectively). The maximum Zn uptake was 3,448.8 and 5,650.4 mg·kg^−1^, respectively (total accumulation of single plants was 59.3 and 63.2 mg, respectively). This indicates that earthworm inoculation can noticeably increase the uptake and transport of Cd, and Zn from the soil environment to the plant biomass by *N. reynaudiana*.

### Subcellular distribution of heavy metals in *N. reynaudiana* affected by the interaction of earthworms and EDTA

In treatments without EDTA and earthworms, Pb, Cd, and Zn were predominantly distributed in the cell wall components of *N. reynaudiana*, followed by soluble components, while only a small amount was found in the nucleus, chloroplast components, and mitochondrial component (Tables 1–3). EDTA treatment promoted the transport of Pb, Cd, and Zn from the F1 cell wall fraction to the F4 vacuole fraction; in particular, Pb in the cell wall component of leaves and roots reached values above 50%. Compared to treatment without EDTA, for the treatment with addition of 5 mmol·L^−1^ EDTA, Pb contents in the cell wall components of leaves and roots were reduced by about 18%, which was only one third of the initial value; the Pb contents in the soluble components of *N. reynaudiana* leaves and roots increased from the initial 30% to above 60%, especially for treatments with earthworms, while the proportion of Pb in the soluble component of roots reached 74.14%.

**Table 1:** Subcellular distribution of Pb^2+^ in *N. reynaudiana* under different concentrations of EDTA with (E) or without earthworms (NE) (mg·kg^−1^).

Pb	Earthworm treatment	EDTA concentration/μmol·L^−1^	F1 (cell wall fraction)	F2 (cell nucleus and/or chloroplast fraction)	F3 (mitochondria fraction)	F4 (soluble fraction)
Leaves	NE	0	5.61 ± 0.72a(52.09)	0.88 ± 0.20a(8.17)	0.75 ± 0.44a(6.96)	3.53 ± 0.95a(32.78)
		2.5	10.4 ± 2.43b(40.34)	1.96 ± 0.40ab(7.60)	1.06 ± 0.12a(4.11)	9.86 ± 1.20ab(3825)
		5	15.87 ± 0.24c(19.90)	3.95 ± 0.06b(4.95)	3.43 ± 0.45b(4.30)	51.49 ± 1.53c(64.57)
	E	0	6.81 ± 0.10ab(50.78)	1.63 ± 0.5ab(12.16)	0.98 ± 0.38a(7.31)	3.99 ± 1.10a(29.75)
		2.5	8.29 ± 0.68ab(22.82)	1.85 ± 0.7a(5.09)	3.18 ± 0.18b(8.75)	20.51 ± 0.59b(56.25)
		5	33.48 ± 3.51d(17.45)	22.59 ± 1.47c(11.78)	7.06 ± 0.68c(3.68)	123.69 ± 16.77d(64.48)
Roots	NE	0	65.57 ± 4.88a(56.45)	5.72 ± 0.27a(4.92)	8.48 ± 0.48b(7.30)	36.39 ± 4.15a(31.33)
		2.5	51.02 ± 3.44a(37.94)	10.14 ± 0.36a(7.54)	3.43 ± 0.38a(2.55)	67.40 ± 5.92b(50.12)
		5	64.76 ± 6.61a(27.57)	8.12 ± 0.92a(3.46)	10.45 ± 0.10bc(4.45)	146.53 ± 14.06c(62.39)
	E	0	65.80 ± 3.96a(52.56)	18.48 ± 3.67b(14.76)	4.81 ± 0.60ab(3.84)	36.11 ± 4.90a(28.84)
		2.5	60.44 ± 8.12a(37.58)	24.39 ± 3.73c(15.17)	3.84 ± 0.20a(2.39)	69.66 ± 0.55b(43.31)
		5	52.84 ± 3.28a(17.12)	14.45 ± 2.76b(4.71)	10.79 ± 0.13c(3.52)	223.88 ± 8.96d(72.39)

F1 represents cell wall, F2 the cell nucleus and/or chloroplast, F3 mitochondria, and F4 soluble constituents. Different letters in the same fraction indicate significant differences among different treatments. Values in parentheses indicate the percentage of Pb in different subcellular components of *N. reynaudiana*. Values followed by different letters are significantly different at *P* < 0.05, as determined via SNK multiple range tests.

**Table 2:** Subcellular distribution of Cd^2+^ in *N. reynaudiana* under different concentrations of EDTA of both with (E) and without earthworms (NE) treatments (mg·kg^−1^).

Cd	Earthworm treatment	EDTA concentration/μmol·L^−1^	F1 (cell wall fraction)	F2 (cell nucleus and/or chloroplast fraction)	F3 (mitochondria fraction)	F4 (soluble fraction)
Leaves	NE	0	1.31 ± 0.34b(45.96)	0.24 ± 0.04a(8.42)	0.27 ± 0.08b(9.47)	1.03 ± 0.03a(36.14)
		2.5	1.64 ± 0.03c(38.86)	0.22 ± 0.00b(5.21)	0.16 ± 0.01a(3.79)	2.20 ± 0.07b(52.13)
		5	0.83 ± 0.07a(22.43)	0.45 ± 0.05c(12.16)	0.13 ± 0.00a(3.51)	2.29 ± 0.11b(61.89)
	E	0	4.41 ± 0.28 f(42.69)	0.62 ± 0.06c(6.00)	0.38 ± 0.06c(3.68)	4.92 ± 1.16c(47.63)
		2.5	3.73 ± 0.27e(29.03)	0.51 ± 0.13bc(3.97)	1.11 ± 0.06e(8.64)	7.50 ± 0.12e(58.37)
		5	2.33 ± 0.09d(22.73)	0.97 ± 0.00d(9.46)	0.72 ± 0.09d(7.02)	6.23 ± 1.05d(60.78)
Roots	NE	0	4.79 ± 0.58c(52.75)	0.51 ± 0.14a(5.62)	0.36 ± 0.01a(3.96)	3.42 ± 0.31a(37.67)
		2.5	3.26 ± 0.25b(34.14)	0.64 ± 0.08a(6.70)	0.34 ± 0.03a(3.56)	5.31 ± 0.09b(55.60)
		5	2.03 ± 0.35a(26.47)	0.57 ± 0.05a(7.43)	0.43 ± 0.03a(5.61)	4.64 ± 0.13c(60.50)
	E	0	2.64 ± 0.18a(25.83)	1.17 ± 0.11b(11.45)	0.81 ± 0.26b(7.93)	5.60 ± 0.27b(54.79)
		2.5	2.35 ± 0.34a(20.52)	1.23 ± 0.07b(10.74)	0.25 ± 0.01a(2.18)	7.62 ± 0.44d(66.55)
		5	2.47 ± 0.55a(31.99)	0.54 ± 0.03b(6.99)	0.68 ± 0.23b(8.81)	4.03 ± 0.88b(52.20)

F1 represents cell wall, F2 the cell nucleus and/or chloroplast, F3 mitochondria, and F4 soluble constituents. Different letters in the same fraction indicate significant differences among the different treatments. Values in parentheses indicate the percentage of Cd in different subcellular components of *N. reynaudiana*. Values followed by different letters are significantly different at *P* < 0.05, as determined via SNK multiple range tests.

**Table 3:** Subcellular distribution of Zn^2+^ in *N. reynaudiana* under different concentrations of EDTA of both with (E) and without earthworms (NE) treatments (mg kg^−1^).

Zn	Earthworm treatment	EDTA concentration/μmol·L^-1^	F1 (cell wall fraction)	F2 (cell nucleus and/or chloroplast fraction)	F3 (mitochondria fraction)	F4 (soluble fraction)
Leaves	NE	0	39.30 ± 0.59a(33.36)	12.00 ± 1.20a(10.19)	8.27 ± 1.43b(7.02)	58.24 ± 5.83a(49.22)
		2.5	48.38 ± 3.68a(28.35)	12.23 ± 0.27a(7.17)	5.17 ± 0.60a(3.03)	104.89 ± 4.00b(61.46)
		5	50.68 ± 4.30a(22.22)	31.15 ± 2.12d(13.66)	3.36 ± 0.60a(1.47)	142.89 ± 3.03c(62.65)
	E	0	72.88 ± 4.40b(27.14)	26.65 ± 4.25c(9.92)	10.19 ± 0.98b(3.79)	158.86 ± 2.46c(59.15)
		2.5	76.70 ± 3.77b(24.20)	19.62 ± 4.68b(6.19)	25.46 ± 1.90c(8.03)	195.21 ± 3.26d(61.58)
		5	81.43 ± 0.45c(18.71)	50.14 ± 5.65e(11.52)	32.75 ± 2.37d(7.52)	270.96 ± 15.19e(62.25)
Roots	NE	0	151.45 ± 11.43a(42.71)	11.08 ± 0.3a(3.12)	24.08 ± 0.23a(6.79)	168.03 ± 5.64a(47.38)
		2.5	101.12 ± 0.64b(24.84)	12.49 ± 3.41a(3.07)	19.87 ± 2.84b(4.88)	273.65 ± 15.03b(67.21)
		5	98.65 ± 3.10b(21.70)	35.61 ± 4.29b(7.83)	25.76 ± 1.73a(5.67)	294.64 ± 5.18c(64.80)
	E	0	120.68 ± 18.36a(26.15)	46.36 ± 2.03a(10.05)	36.14 ± 8.81a(7.83)	258.34 ± 31.67a(55.98)
		2.5	160.35 ± 18.52b(26.43)	46.62 ± 6.99a(7.68)	26.12 ± 1.82a(4.31)	373.58 ± 28.63b(61.58)
		5	113.07 ± 2.86a(21.42)	39.15 ± 1.13a(7.42)	34.81 ± 0.87a(6.60)	340.75 ± 5.14b(64.56)

F1 represents cell wall, F2 the cell nucleus and/or chloroplast, F3 mitochondria, and F4 soluble constituents. Different letters in the same fraction indicate significant differences among different treatments. Values in parentheses indicate the percentage of Zn in different subcellular components of *N. reynaudiana*. Values followed by different letters are significantly different at *P* < 0.05, as determined via SNK multiple range tests.

### Contents and chemical forms of heavy metals in soil under the combined effect of EDTA and earthworms

**Figure 3:**
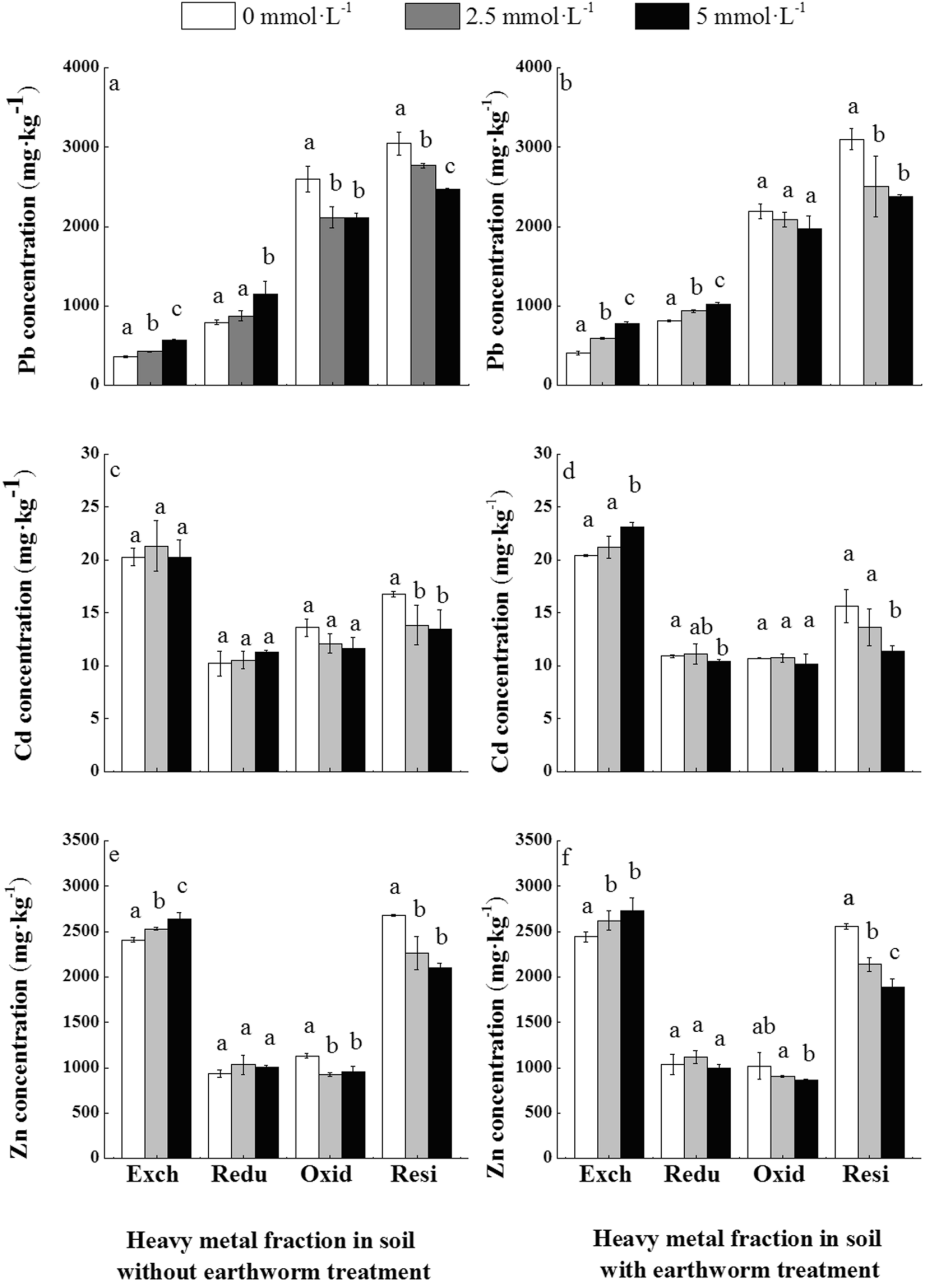
Change of heavy metal fractions in soils under different treatments: left panel represents treatment without earthworms (NE), right panel represents treatment with (E). Exch = Exchangeable fraction; Redu = Reducible fraction; Oxidi = Oxidizable fraction; Resi = Residual fraction. Values followed by different letters are significantly different at *P < *0.05, as determined via SNK multiple range tests.

Lead (Pb) in soil mainly existed in the form of oxidizable and residue fractions (>70%). The exangeable fraction that can be directly used by animals and plants is relativelly smaller (5.34–12.61%) compared to reducible fraction which can potentially affects the environment (11.66–18.2%). After EDTA application, with or without earthworms, the exchageable and reducible Pb fractions increased significantly (17–90% and 10–44%, respectively) whereas the oxidizable and residual Pb fractions decreased (5–19% and 10–23%, respectively) (Fig. 3, Table S1). This suggested that the complexation of EDTA and Pb is facilitated by Pb conversion from organic-bound and residue fractions to exchangeable and reducible fractions, which then increased plant availability.

Zinc (Zn) and cadmium (Cd) mainly occurred as exchangeable and residue fractions, followed by oxidizable and reducible fractions compared to Pb. In treatments without earthworms, and with increasing EDTA concentrations, the exchangeable and reducible Cd and Zn contents increased, while the oxidizable and residual Cd and Zn concentrations decreased.

Furthermore, in treatments with earthworms, exchangeable and reducible Pb, Cd and Zn fractions increased while oxidizable and residual fractions of the same elements decreased.

## Discussion

### Earthworm addition increases *N. reynaudiana* biomass, metal uptake, and transport

Based on the results of previous studies, earthworms can be effectively used to complement soil phytoremediation projects since they support the removal of heavy metals by facilitating interactions between plants and microorganisms^18–20^. Our results show that the presence of earthworms significantly promoted *N. reynaudiana* growth and thus increased its biomass, likely because earthworms improved the conditions of heavy metal contaminated soil, enhance nutrient cycling, increase soil nutrient availability, and as a result, facilitate plant growth^18^. For example, Jusselme *et al*.^21^ reported that in lead-contaminated soils, the presence of earthworms caused a significant decrease in soil pH (by about 0.2), while it increased CEC by 17% and OM by more than 30%. Earthworm activities also increased the activities of N-acetylglucosamidase, β-glucosidase, cellulase, xylanase, alkaline and acid phosphatase, urease, and fluorescein diacetate^21,22^. In addition, our data also indicate that earthworms can promote both uptake and accumulation of heavy metals, especially of Pb and Zn, by *N. reynaudiana*. This is in agreement with most previous studies that suggested an increase of metal availability as a result of earthworm activities in metal-contaminated soils. For instance, earthworm (*Pheretima sp*) inoculation increased plant shoot biomass by 29–83% for *Lolium multiflorum* and by 11–42% for *Brassica juncea* as well as increasing the accumulation of Zn in aboveground parts^19^. A similar study showed that the presence of earthworms (*Pontoscolex corethrurus*) enhanced *Lantana camara* biomass by about 1.5–2-fold and increased the uptake of lead by about 2-3-fold^20^.

Earthworm inoculation significantly increased the uptake of heavy metals by *N. reynaudiana*, most likely because earthworm activity facilitated the heavy metal conversion from stable forms (the residue fraction, the organic fraction, and the Fe-Mn fraction) to available forms (the exchangeable fraction and the water-soluble fraction), which provided favorable conditions for the uptake of heavy metal ions by plant roots. Similarly, when earthworms were used for the remediation of Pb-contaminated soil, in combination with *Lantana camara*, the worms *(Pontoscolex corethrurus*) improved the Pb-availability in the soil around plant roots and promoted Pb uptake^21^. Aghababaei *et al*.^22^ reported that under Cd stress, albeit at relative low concentrations, earthworm activities increased the exchangeable Cd contents in soil and promoted Cd accumulation in the aboveground parts of *Zea mays*. Similarly, Sizmur *et al*.^23^ reported that earthworms mobilized heavy metals such as Cu, Zn, Cd, and Ni in sludge, which decreased the amount of reducing substances, changed the redox state of heavy metals, and thus facilitated heavy metal activation. In treatments with earthworms only, the soil Zn availability increased by 31%, while Cd availability increased by 78–193%^24^. This can be explained as follows: (i) earthworms change the availability of heavy metals in the soil via feeding and excreting; Devliegher and Verstraete^25^ reported that earthworm activity increased the availability of Cr and Co in the soil by digesting and excreting these metals; (ii) earthworm activities have also been reported to change soil pH and thus influence the morphology and transport of heavy metals; the research of Yu^21^ showed that the presence of earthworms (*Pheretima sp*.) reduced the soil pH by 0.2, increased Cd availability, and promoted the Cd uptake of *Lolium multiflorum*; (iii) earthworm activities affect the decomposition of soil organic matter and increase humus and organic acid contents; earthworm secretions contain active groups such as carboxyl and amino groups, which can form complexes with heavy metals, reduce toxic metal levels in soil, and improve the transport of heavy metals from roots to shoots^26^.

### EDTA improves heavy metal availability in the soil and promotes transport and transformation of heavy metals by *N. reynaudiana*

EDTA addition can increase the amount of soluble heavy metals in the soil considerably and promote the uptake of heavy metals by plants^27,28^, which has been confirmed by the findings of our study. In treatments with EDTA, both transport and transformation of Pb, Zn, and Cd by *N. reynaudiana* were effectively promoted. This can be explained by the fact that EDTA mobilized the highly stable Fe-Mn oxides and residue Pb, Zn, and Cd while also increasing the exchangeable and carbonate-bound fractions, thus improving heavy metal availability. Similarly, the research of Grčman *et al*.^15^ showed that following application of 10 mmol kg^−1^ EDTA to the soil, the Pb, Zn, and Cd concentrations of *Brassica rapa* roots increased by 41, 71, and 69%, respectively, compared to the control treatment. The accumulation of Pb, Zn, and Cd by *N. reynaudiana* were 37.9, 10.4, and 56.3% of the total amounts in the soil, respectively. In a different experiment, EDTA increased the Pb concentration of aboveground parts of *Brassica juncea* up to 11,312 mg/kg (accounting for 1.1% of the dry weight); this content was 75-fold that of the Pb concentration in a standard nutrient solution and over 400-fold that of the Pb concentration in plants of the control group^11^. Huang *et al*.^29^ reported that after EDTA entered root cells, it damaged the endodermis and the Casparian strip, which otherwise act as barriers for heavy metals. This finding is supported by the results of our study.

EDTA increased the heavy metal contents in *N. reynaudiana*, and it also changed the distribution of heavy metals in the subcellular components. Heavy metals were mainly distributed in the F1 cell wall and the F4 vacuole components, indicating that cell wall precipitation and vacuolar compartmentation play important roles in the heavy metal uptake of *N. reynaudiana*. This is consistent with previously reported research results^30–32^. However, after EDTA treatment, the proportions of Pb, Zn, and Cd significantly decreased in the cell wall of *N. reynaudiana*, while those in the F4 vacuolar component significantly increased, indicating that EDTA lowered the cell wall affinity to heavy metals. This could be explained by the negatively charged carboxyl and polysaccharide groups within the cell wall, which favored the uptake of positively charged metal cations. Similar research has shown that EDTA has the ability to reduce cell wall uptake of heavy metals via formation of Me-EDTA complexes that enter the cells and are stored in the vacuoles, thus improving the proportion of heavy metals in the soluble component and to some extent, mitigating heavy metal toxicity for plants^32^, which was also reported by He *et al*.^31,33^. In *Chamaecytisus palmensis*, Pb mainly accumulated in the roots, while EDTA chelated Pb was mainly concentrated in aboveground parts, especially in the chloroplast, pit membrane, and the plasmodesmata^27^.

### Earthworms and EDTA in combination improved the remediation efficiency of *N. reynaudiana*

Our results indicate that the combined application of EDTA and earthworms had a more significant effect on Pb uptake and accumulation by *N. reynaudiana* compared to a treatment that only used earthworms or EDTA. We therefore infer that the combined application of earthworms and EDTA mobilized heavy metals in the soil, thus increasing the amounts of available heavy metals. In addition, synergetic effects may also play a role, since earthworm activities change the soil physical structure and increase soil porosity^33^. Leveque *et al*. (2014)^34^ showed that polluted soils were strongly bioturbated in the form of vertical burrows and near-horizontal burrows, thus increasing soil macroporosity.

According to Stampoulis *et al*.^35^, these burrows are preferred pathways for plant roots and may enable the roots to penetrate the soil more rapidly and deeply, which could potentially facilitate heavy metal uptake that were mobilized by EDTA. Furthermore, according to Farenhorst *et al*.^36^, *Lumbricus terrestris* builds permanent vertical burrows and drops casts at the soil surface. This species also typically lines its burrows with cast material, which increases metal leaching and favors contact with plant roots.

## Conclusion

Both EDTA and earthworms can increase the amounts of heavy metals that can be accumulated by *N. reynaudiana*. Combined application of earthworms and EDTA optimized the effect on the uptake and accumulation of heavy metals by *N. reynaudiana*. Therefore, results in this study confirmed our hypothesis that earthworm activities improve soil properties, increase soil available nutrients, improve *N. reynaudiana* biomass in contaminated soil, and increase soil porosity via soil disturbance, promoting the mixing between EDTA and rhizosphere soil, and improving the ability of EDTA to mobilize heavy metals. Our results provide a theoretical basis to further the application of earthworms and EDTA for the improvement of phytoremediation techniques and for the increase of plant production in heavy metal contaminated soils.

## Materials and Methods

### Experimental materials

The sampling area was located in the surrounding farmlands of the Huangbei field in the lead-zinc mining area of Miaoqian Town, Liancheng County, Longyan City, Fujian Province (N 25°21′ and E 116°43′). Soil samples were taken from the 0–20 cm soil layer. The chemical soil properties were measured: pH 5.2, total phosphorous (P) content 0.31 g·kg^−1^, total potassium (K) content 5.54 g·kg^−1^, available P content 1.18 mg·kg^−1^, and available K content 41 mg·kg^−1^. The contents of Pb, Cd, and Zn were 6.16, 0.03, and 6.42 g·kg^−1^, which exceeded the Chinese Soil Quality threshold values. The collected soil samples were air-dried and all gravel as well as debris of plants and animals were removed. Subsequently, the soil samples were ground and passed through a 2-mm sieve. After mixing and equilibrium for two weeks, 2-kg subsamples were placed into individual plastic pots.

### Research method

After the soil samples were equilibrated for one week, equally sized *N. reynaudiana* plants were transplanted into the contaminated soil, using six plants per pot. For the treatment containing earthworms, 15 earthworms (*Eisenia fetida*), gut-purged on filter paper for 24 h, were added to each pot. According to the OECD methods^13^, earthworms were acclimatized to the experimental conditions for at least 7 d prior to exposure (temperature, light, soil moisture, etc.). Only healthy earthworms, weighing between 200 and 600 mg, with a well-developed clitellum, were introduced. To ensure earthworm survival during the experimental period, worms were weekly fed with a dried mixture of cattle manure (0.5 g per earthworm). After cultivation for four weeks, according to the method of Zhao *et al*.^14^, 100 ml of EDTA was applied once at different concentrations (0, 2.5, and 5 mmol·kg^−1^ of deionized water). EDTA was no added to the control group, resulting in two treatment groups: i) control groups without earthworm inoculation and without EDTA addition, without earthworm inoculation +2.5 mmol·kg^−1^ EDTA, and without earthworm inoculation +5 mmol·kg^−1^ EDTA; ii) control groups with earthworm inoculation and without EDTA addition, with earthworm inoculation +2.5 mmol·kg^−1^ EDTA, and with earthworm inoculation +5 mmol·kg^−1^ EDTA. Five days after EDTA application, *N. reynaudiana* and soil samples were harvested, using four replicates per treatment. Throughout the incubation period, the plants were cultured in an incubator at 30 000 LX, 16 h of illumination at 25 °C, and 8 h of darkness at 22 °C. During plant growth, water was added to maintain soil moisture at about 60% of field capacity.

### Measurement of the total heavy metal contents in soil

Part of the soil samples were ground, passed through a 0.149 mm-sieve, and placed into a 30-ml Teflon crucible. Soils (0.5 g) were digested with a mixture of concentrated HF–HClO_4_–HNO_3_ on a hot plate. The digested solution was then cooled, filtered, and finally diluted to 25 mL^36,37^. The concentration of heavy metals (Pb, Zn, and Cd) was measured via inductively coupled plasma-atomicemission spectroscopy (ICP-AES, Optima 8000 Perkin Elmer Corporation). Quality assurance and control (QA/QC) included a procedural blank, duplicate analysis, and standard reference materials. The accuracy was calculated from the relative error of the certified values of standard reference materials and was below 10%. The relative standard deviation (RSD) of duplicate samples was below 5%.

### Speciation of heavy metals with the modified BCR sequential extraction procedure

Soil samples were extracted before the experiment and 5 d after adding the chelating agent. The BCR method was employed to sequentially extract the availability of heavy metals in the soil^15,38^, performing the following steps: i) Weak acid extractable fraction: 1 g of sample was weighed and 40 ml of 0.11 mol·L^−1^ acetic acid (HAc) was used as extractant. ii) Reducible state: The reducible elements were obtained from the second extraction, which was performed using 0.1 M hydroxylamine hydrochloride (NH_2_OH·HCl) at pH 2.0. iii) Oxidizable fraction: During the third extraction, the oxidizable fraction was obtained and 1 M ammonium acetate (CH_3_COONH_4_, pH 2) was used after oxidation with 8.8 M H_2_O_2_. iv) Residue fraction: The residue was digested with HNO_3_, HCl, and HClO_4_ and then diluted to 50 ml. The solutions were directly measured via ICP-AES, and deionized water was used to substitute samples. A blank solution was prepared based on the methods mentioned above.

### Accumulation and subcellular distribution of heavy metals *in N. reynaudiana*

After the experimental period, the roots and aboveground parts of washed plants were separated, air-dried, oven-dried with desiccation for 30 min at 105 °C, and dried to a constant weight at 75 °C^16^. Samples were digested with HNO_3_/HClO_4_ (3:1, v/v) solution^16^. The digested samples were dissolved in deionized water (25 ml) and determined via ICP-AES. For quality control, 10 repeated measurements were conducted with standard Pb solution that was provided by the Institute of Geophysical and Geochemical Exploration, China^16,38^.

The method of differential centrifugation was adopted to separate different cellular components^1,17^. After the experiment, 0.2 g of fresh sample were weighed and 20 mL of extract (0.25 mol·L^−1^ sucrose +50 mmol·L^−1^ Tris-HCl buffer solution, (pH 7.5) +1 mmol·L^−1^ dithioerythritol) were added; liquid nitrogen was used to grind the homogenate, which was then centrifuged for 1 min at 300 r·min^−1^ under refrigerated conditions; the obtained precipitate consisted of cell wall components (F1). The supernatant was centrifuged for 15 min at 2,000 r·min^−1^; the obtained precipitate consisted of nuclear components (F2). The resulting supernatant was centrifuged for 20 min at 1,0000 r·min^−1^ to separate mitochondrial components (F3); the obtained supernatant consisted of soluble components containing ribosomes and mainly vacuoles (F4)^17^. All operations were conducted at 4 °C and all components were measured via ICP-AES after digestion as described above.

### Data processing

We used the following equations:

Data of biomass, heavy metal accumulation, and subcellular distribution of heavy metal in *N. reynaudiana* were analyzed using two-way ANOVA, followed by Student-Newman-Keuls (SNK) for post-hoc analysis. A value of *P* < 0.05 was considered as statistically significant.

One-way ANOVA was used to compare heavy metals fraction in soil, total heavy metal contents in *N. reynaudiana*, and heavy metal accumulations in earthworms. Means that exhibited significant differences were compared via SNK significance test at a 0.05 level of probability. A paired sample t-test was used to compare the total heavy metal contents in *N. reynaudiana* between treatments with or without earthworms. The graphical representations were obtained with Origin 9.0.

All data were processed using the statistical package SPSS (SPSS 19.0 for Windows, SPSS Inc., Chicago, Illinois, USA); the presented values represent the means of four replicates.

Bioconcentration Factor (BCF) = heavy metal concentration of roots/heavy metal concentration of soil; Translocation Factor (TF) = heavy metal concentration of aboveground parts/heavy metal concentration of roots; Extraction Efficiency (EE) = (total extraction amount of heavy metals of aboveground parts/total heavy metal contents of soil) × 100%.

## Electronic supplementary material

Supplementary Dataset 1
