# Supplementary material for: Improvement of the phytoremediation efficiency of Neyraudia reynaudiana for lead-zinc mine-contaminated soil under the interactive effect of earthworms and EDTA
Source: Sci Rep. 2018 Apr 23;8:6417. doi: 10.1038/s41598-018-24715-2 (PMC5913105; doi:10.1038/s41598-018-24715-2)
Supplement: Supplementary file 1 — Supplementary Dataset 1 [file 41598_2018_24715_MOESM1_ESM.docx]

**Improvement of the phytoremediation efficiency of *Neyraudia reynaudiana* for lead-zinc mine-contaminated soil under the interactive effect of earthworms and EDTA**

Ying Li^abc^, Jiewen Luo^abc^, Jiaoda Yu^abc^, Lidan Xia^abc^, Chuifan Zhou^abc^ *, Liping Cai^abc^, Xiangqing Ma^abc^

a. College of Forestry Fujian Agriculture and Forestry University, Fuzhou China

b. Co-Innovation Center For Soil and Water Conservation in Red Soil Region of the Cross-Straits.

c. Red Soil Hilly Ecosystem Positioning Observation Station in Changting of Fujian

* Correspondence:zhouchuifan@163.com;

**Fig. S1** Total amounts of heavy metals accumulated by *Neyraudia reynaudiana* in response to different concentrations of EDTA. Notes: different upper- and lowercase letters represent significant differences between treatments with (E) and without earthworms (NE) at different EDTA concentrations (*P <* 0.05), as determined via SNK multiple range tests. The asterisk denotes the significance of difference between E and NE treatments under the same EDTA concentrations (*, *P <* 0.1; **, *P <* 0.05; and ***, *P <* 0.01), as determined via paired-samples t-test.

**Fig. S2** Accumulation of heavy metals in earthworms with or without EDTA treatment. Different lowercase letters represent significant differences between with (E) and without earthworms (NE) treatments at different concentrations (*P <* 0.05), as determined via SNK multiple range tests.

**Table S1** Earthworm mortality with or without EDTA treatment. Notes: different lowercase letters represent significant differences between with (E) and without earthworms (NE) treatments at different concentrations (*P <* 0.05), as determined via SNK multiple range tests.

**Table S2** Percentages of different metal fractionations in the soil (%) under different concentrations of EDTA for with (E) and without earthworms (NE) treatments.

**Table. S3** Bioconcentration factors (BCFs), translocation factor (TFs), and extraction efficiency (EE) under different treatments.

Fig. S1


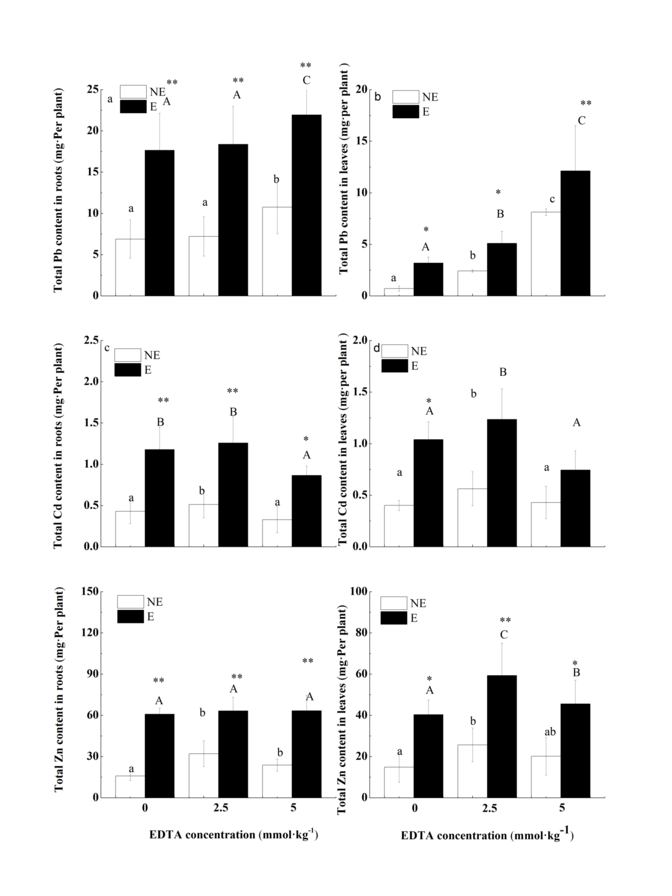


Fig.S2

Table S1

| EDTA concentration | Mortality rate (%) |
| --- | --- |
| 0 | 83.3±12.3a |
| 2.5 | 44.2±23.4b |
| 5 | 18.6± 14.3c |

Table S2

| Metal | Earthworm treatment | EDTA concentration  μmol·L^-1^ | Exchangeable  fraction | Reducible  fraction | Oxidizable  fraction | Residual  fraction |
| --- | --- | --- | --- | --- | --- | --- |
| Pb | NE | 0 | 5.34 | 11.66 | 38.22 | 44.78 |
|  |  | 2.5 | 6.92 | 14.15 | 34.22 | 44.72 |
|  |  | 5 | 9.10 | 18.20 | 33.52 | 39.18 |
|  | E | 0 | 6.26 | 12.49 | 33.64 | 47.62 |
|  |  | 2.5 | 9.65 | 15.32 | 34.12 | 40.91 |
|  |  | 5 | 12.61 | 16.61 | 32.04 | 38.73 |
| Cd | NE | 0 | 33.31 | 16.80 | 22.37 | 27.52 |
|  |  | 2.5 | 36.86 | 18.25 | 20.96 | 23.93 |
|  |  | 5 | 35.78 | 19.94 | 20.53 | 23.75 |
|  | E | 0 | 35.40 | 18.92 | 18.60 | 27.09 |
|  |  | 2.5 | 37.36 | 19.64 | 18.93 | 24.06 |
|  |  | 5 | 41.96 | 19.00 | 18.42 | 20.61 |
| Zn | NE | 0 | 33.67 | 13.05 | 15.81 | 37.46 |
|  |  | 2.5 | 37.48 | 15.27 | 13.71 | 33.54 |
|  |  | 5 | 39.30 | 15.04 | 14.28 | 31.39 |
|  | E | 0 | 34.63 | 14.69 | 14.43 | 36.26 |
|  |  | 2.5 | 38.66 | 16.42 | 13.36 | 31.56 |
|  |  | 5 | 42.05 | 15.44 | 13.38 | 29.13 |

Table S3

| Metal | Earthworm  treatment | EDTA  concentration/μmol·L-1 | BCF | TF | EE  (%) |
| --- | --- | --- | --- | --- | --- |
| Pb | NE | 0 | 0.16 | 0.07 | 0.07 |
|  |  | 2.5 | 0.18 | 0.22 | 0.232 |
|  |  | 5 | 0.30 | 0.37 | 0.790 |
|  | E | 0 | 0.24 | 0.11 | 0.31 |
|  |  | 2.5 | 0.27 | 0.18 | 0.50 |
|  |  | 5 | 0.36 | 0.35 | 1.18 |
| Cd | NE | 0 | 2.04 | 0.59 | 8.01 |
|  |  | 2.5 | 2.59 | 0.72 | 11.24 |
|  |  | 5 | 1.88 | 0.64 | 8.58 |
|  | E | 0 | 3.27 | 0.56 | 20.78 |
|  |  | 2.5 | 3.75 | 0.64 | 24.72 |
|  |  | 5 | 2.88 | 0.54 | 14.89 |
| Zn | NE | 0 | 0.35 | 0.60 | 1.39 |
|  |  | 2.5 | 0.76 | 0.53 | 2.39 |
|  |  | 5 | 0.64 | 0.41 | 1.88 |
|  | E | 0 | 0.79 | 0.42 | 3.77 |
|  |  | 2.5 | 0.88 | 0.61 | 5.54 |
|  |  | 5 | 0.99 | 0.45 | 4.25 |
